# A stacked CNN and random forest ensemble architecture for complex nursing activity recognition and nurse identification

**DOI:** 10.1038/s41598-024-81228-x

**Published:** 2024-12-30

**Authors:** Arafat Rahman, Nazmun Nahid, Björn Schuller, Md Atiqur Rahman Ahad

**Affiliations:** 1 University of Virginia, Charlottesville, USA; 2 Kyushu Institute of Technology, Kitakyushu, Japan; 3 Technical University of Munich, Munich, Germany; 4 Department of Computer Science and Digital Technologies, University of East London, London, UK

**Keywords:** Nursing activity recognition, User identification, Human activity recognition, Data augmentation, Feature engineering, Deep learning, Ensembles, CNN, Quality of life, Occupational health

## Abstract

Nursing activity recognition has immense importance in the development of smart healthcare management and is an extremely challenging area of research in human activity recognition. The main reasons are an extreme class-imbalance problem and intra-class variability depending on both the subject and the recipient. In this paper, we apply a unique two-step feature extraction, coupled with an intermediate feature ‘Angle’ and a new feature called mean min max sum to render the features robust against intra-class variation. After intermediate and final feature extraction, we use an ensemble of a random forest classifier and a stacked convolutional neural network (S-CNN) model to detect activities and users. Unlike traditional CNN, the S-CNN takes the input feature channels in separate pathways with equal importance, which makes it robust to intra-class variation and produces accurate results. We apply this method to two benchmark open-source nurse care activity data sets. Our algorithm is robust enough to recognize both activity and user (Nurse) simultaneously. During the recognition process, this algorithm automatically finds the important features in the data set. Using this algorithm, the highest testing accuracies were achieved for activity recognition on the two (publicly available in IEEE DataPort) benchmark data sets: The CARECOM Nurse Care Activity (70.6% accuracy) and the Heiseikai Nurse Care Activity data set (85.7% accuracy). Moreover, the highest accuracy achieved for user identification on Data Set 1 and Data Set 2 is 78.2% and 92.7%, respectively.

## Introduction

Human activity recognition (HAR) has been one of the most prevailing and persuasive research topics in different fields in the course of the last few decades. HAR aims to comprehend individuals’ regular activities by looking at bits of knowledge accumulated from individuals and their encompassing living environments. Although there are various applications, the broad objective of the majority of the research works in human activity identification is the remote monitoring of the consistent activities of individuals, such as of pregnant females, elderly, and medical clinic patients. It allows to have a 24-h checking or assessment to allow for omnipresent well-being and health supervision^1^. The worldwide middle age has expanded from 21.5 years in 1970 to more than 30 years in 2022^2^. If we take a gander at the World Population Aging Report 2022, we will locate that the number of people aged 65 or over in 2022 is 771 million everywhere in the world. It is expected to be 1600 million within 2050^3^. So, the demand for nursing care is increasing drastically as a fundamental part of clinical consideration in an assortment of settings. The exercises that medical attendants perform regularly straightforwardly sway the health and well-being of patients^4^. So, nurse care activity recognition can benefit medical services in many ways: (1) quality nursing behavior and positive patient outcomes can be reinforced, (2) instances of negligent or substandard care can be identified and addressed, (3) unnecessary activities and excessive workload can be reduced, and (4) duties can be distributed based on their efficiency in performing tasks.

Despite the significance of nursing activity recognition as a specialized domain within HAR, it presents unique challenges that distinguish it from general HAR. The primary factors contributing to these challenges are:

- Diverse and complex activities: Nursing activities are often more diverse and complex than everyday activities. They involve a wide range of tasks, from simple tasks like walking to complex ones like patient care and medication administration. This diversity makes it difficult to capture all the nuances of these activities^5^.
- Contextual factors: Nursing activities are often influenced by the environment, patient conditions, and specific care plans. These contextual factors can significantly impact the sensor data and make it harder to accurately recognize activities^6^.
- Sensor noise and variability: Sensors deployed can be noisy and prone to errors, especially in real-world settings.This noise can affect the accuracy of activity recognition algorithms^7^.
- Intra-class Variability: Unlike other activity recognition where the users are doing an activity by themselves, nurses usually perform most activities for a patient. So, intra-class variability can be seen, which depends not only on the subject but also on the receiving patient.
- Data Collection: Also, in real-world settings, there is a high chance of getting missing data and labels during experiments as nurses are busy at work. Also, class-imbalance is often observed in such data, as there is no particular cycle to follow while assisting the patients; some activities are required to be performed more frequently than others depending on the patient’s condition. Moreover, problems like non-uniform sampling rate, presence of redundant data, the absence of a precise timestamp, and many more make the work difficult and often require multiple sensor data^8–10^, which requires high cost.

Hence, due to the intricate nature of nursing care activities, the significant cost associated with data collection, and the scarcity of labeled activity data, the development of sensing-based nurse care activity recognition systems has been limited. Additionally, many state-of-the-art HAR techniques struggle to achieve satisfactory performance when applied to nursing care data, primarily due to the unique characteristics and contextual nuances of these activities.

To solve the problem of high cost and being unaccustomed to the data collection setup, smartphones can be a suitable platform for detection, as they are widely used and equipped with a variety of sensors. They consist of an inertial measurement unit (IMU), which usually includes an accelerometer and, in some cases, a gyroscope. For long-term monitoring of human movement use of the accelerometer is increasing rapidly^11^. Such accelerometers typically lead to good results in the detection of physical activity, which generally needs very little computing power^12^. Though this bears many benefits, there are not many works based on accelerometer data only for complex activity recognition like nurse care. Because of the frequency response, dynamic range, and sensitive axis, we work only with accelerometer data in this research.

We have identified several key gaps in the literature: firstly, the need for simultaneous recognition of activities and users, which is not adequately addressed by existing single-focus methods. Secondly, traditional CNNs often fail to capture complex patterns in nursing activities, and thirdly, the integration of traditional machine learning with deep learning approaches remains superficial without leveraging their complementary strengths. There are five main contributions of this paper to address the research gaps:

- We introduced a two-step feature extraction method that significantly enhances the simultaneous recognition of activities and users.
- We also proposed a novel feature, MSUM, tailored for complex activities in nursing, enhancing the model’s effectiveness.
- We proposed a Stacked CNN (S-CNN) approach to overcome the limitations of traditional CNN by better capturing nuanced patterns of nurse care activity recognition.
- We introduce angle as an intermediate feature and by using it, we leverage its analytical strength in both S-CNN and classical machine learning models, enhancing model robustness.
- Lastly, we propose an ensemble method that synergistically combines the strengths of classical and deep learning approaches, outperforming each when used independently.

To the best of our knowledge to date, this type of real-life implementable system has not been developed, yet. Also, to make the proposed system more efficient, we ease on the class-imbalance problem. We work on two dedicated nurse care data sets – (1) the CARECOM Nurse Care Activity data set^13–15^; and (2) the Heiseikai Nurse Care Activity data set^16,17^. The reasons behind choosing these are that the data collected feature both lab and real-world data. Further, as the second data set mainly focused on the real-time challenge, data here are highly imbalanced, have missing labels, redundant values, errouneous time stamps, non-uniform sampling rate, and further more. Likewise, by working with these data sets, it is possible to achieve a result that can be considered near real-time.

The rest of the paper is organized as follows: In Sect. “Related works”, appropriate activity recognition works related to this topic are identified. Then, previous notable works and research gaps are presented. In Sect. “Data sets”, a brief overview of the data sets is given. In Sect. “Methodology”, our proposed method is elaborately described. In Sect. "Result and discussion", the achieved results from different algorithms are illustrated including an in-depth analysis based on our chosen model. Finally, in Sect. "Conclusion and future work", the conclusion and future work are provided.

## Related works

A considerable amount of works have been done based on smartphone sensors and accelerometer data in the field of human activity recognition^18–21^. Also, in the medical field, some notable research has been carried out on activity recognition^22–25^. Recognizing clinical activities has various implications for continuous control among others of heart attack patients, pregnant women, and the elderly. In this aspect, by observing nursing data, we can obtain significant information about the patients and enable a better health facility. By keeping that in mind, two “Nurse Care Activity Recognition Challenges” have been organized to develop efficient systems to identify nurses’ activity accurately. The main focus of the participants of these challenges were to identify activities with higher accuracy^26–37^. In the first challenge, the authors of^28^ used features extracted from motion capture and meditag sensors with a K-Nearest Neighbor (KNN) classifier achieving 87% cross-validation accuracy for Data Set 1. The authors of^26^ used a spatio-temporal graph convolutional network (ST-GCN) reaching 57% leave-one-subject-out cross-validation accuracy. The authors of^37^ extracted different features and compared different machine learning algorithms for identifying 12 activities of Data Set 2. They reported that they achieved the highest accuracy using KNN.

Though in these challenges, activity recognition is the main focus, along with the user identification, it renders the system more robust and efficient. This comes, as likewise we not only have the information about how the work is executed, but also who works better and how we should improve the care facility. In the work^38^, the authors have designed a user identification system along with a cooking activity recognition system. The main difference in our work is that we are using a single system to identify both the user and the activity.

Data Set 2 has a very high class-imbalance. This imbalance results in the real world due to an uneven data distribution in the collected data because of biased sampling and measurement errors. To solve it, there are several approaches like collecting more data, changing performance metrics, resampling data, generating synthetic data points, or trying different algorithms. Among these, resampling and generating synthetic data points or data augmentation are commonly used. Though data augmentation is widely used in image and sound-related works, very little work has been done on wearable sensor-based time-series data augmentation. Many studies in different areas have used interpolation for missing data^39,40^.

The authors of^41^ compared different data augmentation techniques and their combination such as Jitter, scale, crop, rotation, permutation, time warping, or magnitude warping on the wearable sensor data for Parkinson’s disease classification and reported that the combination of rotation, permutation, and time warping achieved the highest accuracy.

The authors of^42^ proposed a data augmentation method for sensor-based gait data, which is composed of two algorithms named arbitrary time deformation (ATD) and stochastic magnitude perturbation (SMP). Then, they classified different gaits using convolutional neural networks. In^43^, the authors proposed an augmentation technique on the spectrogram-based feature space and then classified different simple activities using a long short term memory (LSTM) network. Further, the authors of^44^ proposed a novel data augmentation method, which is based on sub-optimal time-warping, where new class boundaries can be created using the newly generated data. The authors of^45^ created an augmentation algorithm for sensor signals that preserves the labels of the augmented data, and increases the accuracy.

The authors in^46^ proposed a method based on an adversarial autoencoder for handling missing sensory features and synthesizing realistic samples. The main difference between these works and our work is that we not only use augmentation to increase accuracy and reduce overfitting, but also to reduce the class-imbalance by generating new samples for the minority classes automatically. Moreover, in our system, we can identify users simultaneously with the activity they are performing.

## Data sets

**Fig. 1:**
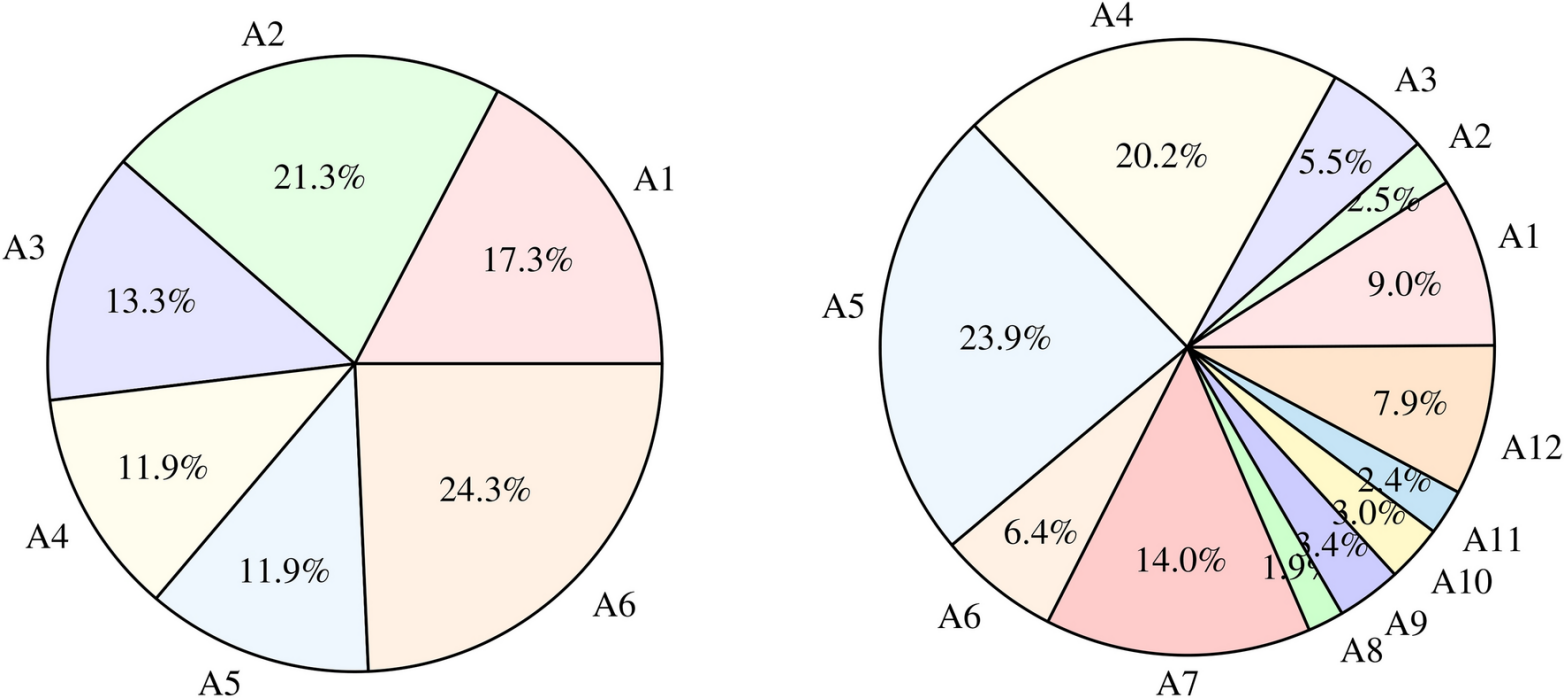
Distribution of recorded minutes of each activity in *Data Set 1* (CARECOM Nurse Care Activity data set): A1. Vital Signs Measurements, A2. Blood Collection, A3. Blood Glucose Measurement, A4. Indwelling Drip Retention and Connection, A5. Oral Care, and A6. Diaper Exchange and Cleaning of the Area; and *Data Set 2* (Heiseikai Nurse Care Activity data set): A1. Guide (from the front), A2. Partial Assistance, A3. Walker, A4. Wheelchair, A5. All Assistance, A6. Partial Assistance (from the front), A7. Partial Assistance (from the side), A8. Partial Assistance (from the back), A9. To Supine Position / To Right Lying Position, A10. To Left Lying Position, A11. Lower Body Lifting, and A12. Horizontal Movement.

Both data sets were recorded in the Smart Life Care Unit of the Kyushu Institute of Technology, Japan^47^. These are publicly available in IEEE DataPort. The brief descriptions of these data sets are given below:

1. Data Set 1: In the CARECOM Nurse Care Activity data set^13–15^, data were collected from the Motion Capture, Meditag, and accelerometer sensor of the Freetel Priori 3 smartphone carried in the right chest pocket of nurses. We only use the accelerometer in our study. In this experiment, 8 subjects participated (all are Japanese). The training data set consists of 6 users’ data, and the test data set consists of 2 different users’ data on different days.
  Each participant performed 5 repetitions of each activity, yielding about 240 activity sequences and 407 recorded minutes. The six performed activities are: A1. Vital Signs Measurements, A2. Blood Collection, A3. Blood Glucose Measurement, A4. Indwelling Drip Retention and Connection, A5. Oral Care, and A6. Diaper Exchange and Cleaning of the Area.
  Even though 40 samples (5 per user) of each activity were collected, the duration of the activities is not the same, so the time distribution is not equal. We show the distribution of the duration of each activity in Fig. 1. The sampling rate of this data is 4 Hz.

(2) Data Set 2: In the Heiseikai Nurse Care Activity data set^16,17^, along with the recording in the Smart Life Care Unit (Lab Data), data were also collected in a Care Facility in Japan (Field Data). In the lab, 2 subjects participated who are professional nurses. In the real field, 47 subjects participated in the experiment, however, data of 9 nurses are free for usage. In the real field, all of them are professional nurses and Japanese citizens.
  The training data set consists of 8 users’ (2 lab users and 6 field users) data, whereas the test data set consists of 3 different field users’ data on different days. The activities can be categorized into 3 principal types: Help in Mobility, Assistance in Transfer, and Position Change. These activities are further divided into 12 classes such as: A1. Guide (from the front), A2. Partial Assistance, A3. Walker, A4. Wheelchair, A5. All Assistance, A6. Partial Assistance (from the front), A7. Partial Assistance (from the side), A8. Partial Assistance (from the back), A9. To Supine Position / To Right Lying Position, A10. To Left Lying Position, A11. Lower Body Lifting, and A12. Horizontal Movement.
  Data were collected from a smartphone accelerometer, which was attached to the right arm using the armband. The sampling rate of the data is 60 Hz. This data set is highly imbalanced in activity labels which can be seen in Fig. 1.

## Methodology

**Fig. 2:**
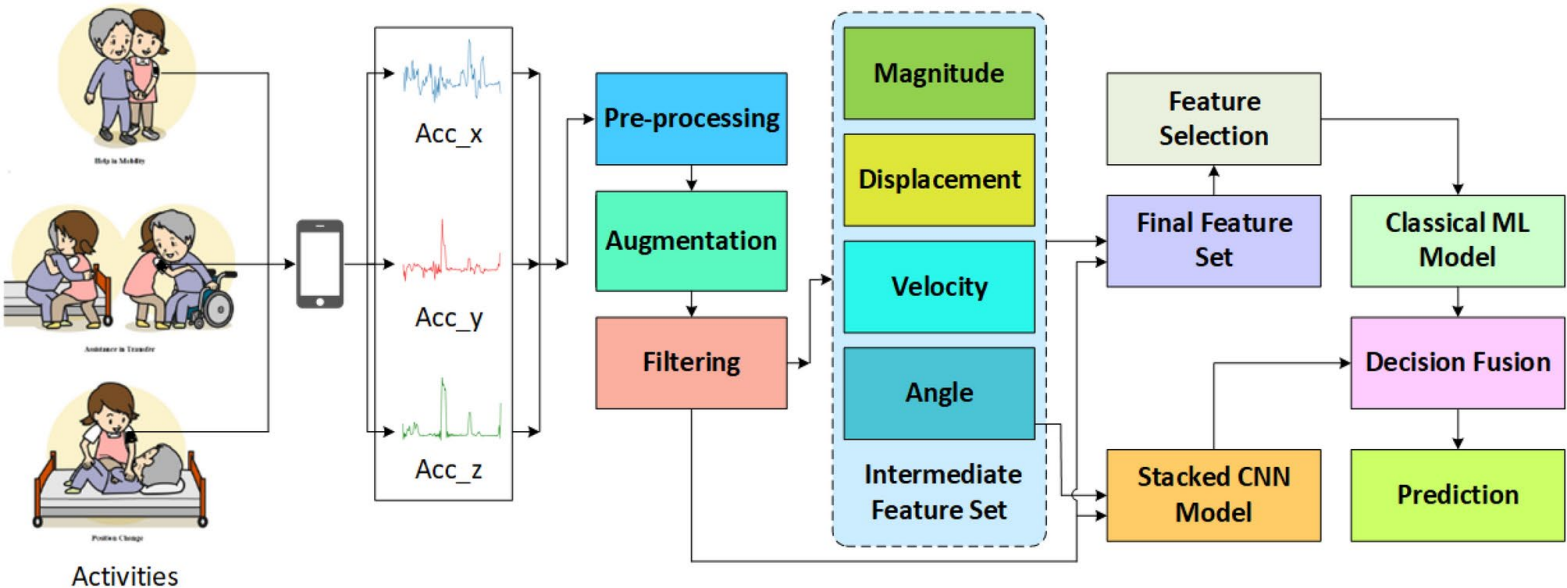
Flow diagram of the nurse care activity recognition system where a ‘classical’ machine learning (ML) model takes the Acc_xyz, magnitude, displacement, velocity, and angle as intermediate features, and a Stacked CNN takes Acc_xyz and Angle as intermediate features. After the prediction of the classical ML model and the stacked CNN, the decision is fused at last stage to make an ensemble model.

In this section, we will formulate the problem and discuss our proposed method in detail. The raw data of Data Set 1 at time step *n* can be expressed as $d_{n}=\{s_{n}, et_{n}, \alpha _{xn}, \alpha _{yn}, \alpha _{zn}\}$, where, $s_{n}$ represents the segment ID; $et_{n}$ represents the elapsed time; $\alpha _{xn}$, $\alpha _{yn}$, and $\alpha _{zn}$ represent the raw acceleration signal samples of the x, y, and z axes, respectively. The raw data of Data Set 2 has a slightly different format and can be expressed as $d_{n}=\{t_{n}, \alpha _{xn}, \alpha _{yn}, \alpha _{zn}\}$, where, $t_{n}$ represents a time stamp and the other parts remain the same as for Data Set 1. The activity labels and user IDs are stored in other files. For Data Set 1, the format of the user ID and the activity ID at time step *n* can be expressed as $l_{n}=\{u_{n}, s_{n}, a_{n}\}$ and for Data Set 2, these can be expressed as $l_{n}=\{u_{n}, a_{n}, st_{n}, ft_{n}\}$, where, $u_{n}$ represents the user ID; $s_{n}$ represents the segment ID; $a_{n}$ represents the activity ID; $st_{n}$; and $ft_{n}$ represent the start and finish time of an activity. For activity recognition, we have to design a machine learning classifier that relates raw data $\alpha _{n}$ with the activity ID $a_{n}$ using the function $A_{\psi}: \alpha _{n} \rightarrow a_{n},$ where, $\psi$ represents the weight of the classifier. For user identification, we similarly need to design a machine learning model that relates raw data $\alpha _{n}$ with the user ID $u_{n}$ using the function $I_{\omega }: \alpha _{n} \rightarrow u_{n},$ where, $\omega$ is the weight of the model. To design this system, we apply several steps including data pre-processing, data segmentation, data augmentation, feature extraction, and feature selection. All of these steps are discussed in the following subsections in detail. Figure 2 shows the whole process of the activity recognition. For user identification, the system remains the same except that instead of activity labels, the user IDs are exploited during training.

### Data pre-processing

Data Set 1 was pre-processed by the dataset provider and well organized, whereas Data Set 2 had not been pre-processed by the dataset provider. Moreover, the Data Set 2 has miss-matched time stamps and labels. So, pre-processing and cleaning are necessary for Data Set 2. For this purpose, at first, all the timestamp formats of data and label files are changed to a standard format, and all data rows are sorted according to timestamp. Finally, all the data samples are merged with labels such that $s_{nd} = s_{nl}$ for Data Set 1 and $st_{n}<= t_{n} <= ft_{n}$ for Data Set 2, where, $s_{nd}$ represents the segment ID of the data sample; $s_{nl}$ represents the segment ID of the label; $st_{n}$ and $ft_{n}$ represent the start and finish time of an activity; $t_{n}$ represents the timestamp of a data sample. The same procedure has been followed while merging with user IDs. During data collection, some data values are lost due to device malfunction and for other reasons. These missing values are imputed by the mean values of their corresponding columns.

### Data augmentation algorithm

Our explored data sets are open access/publicly available in IEEE DataPort (links are provided at the end of the paper). In both nurse care activity data sets, some of the activities are done rarely by nurses in everyday life. This creates a huge class imbalance in both data sets. Moreover, the collection of this type of activity data is arduous and cumbersome which makes it difficult to train any machine learning model. A classifier can easily overfit due to this limited amount of data. Also, due to huge class imbalance, a classifier easily becomes biased towards the majority class and struggles to learn the minority classes. For these reasons, we use a data augmentation algorithm for the targeted complex nursing activity. This algorithm takes the training segments of both Data Set 1 and 2 and enlarges the data sets up to a desired number of segments based on the user input. It augments the data carefully without changing the actual labels and retains the Spatio-temporal relationship or principal properties of the original signal so that the distinctiveness of the activities increases.

**Fig. 3:**
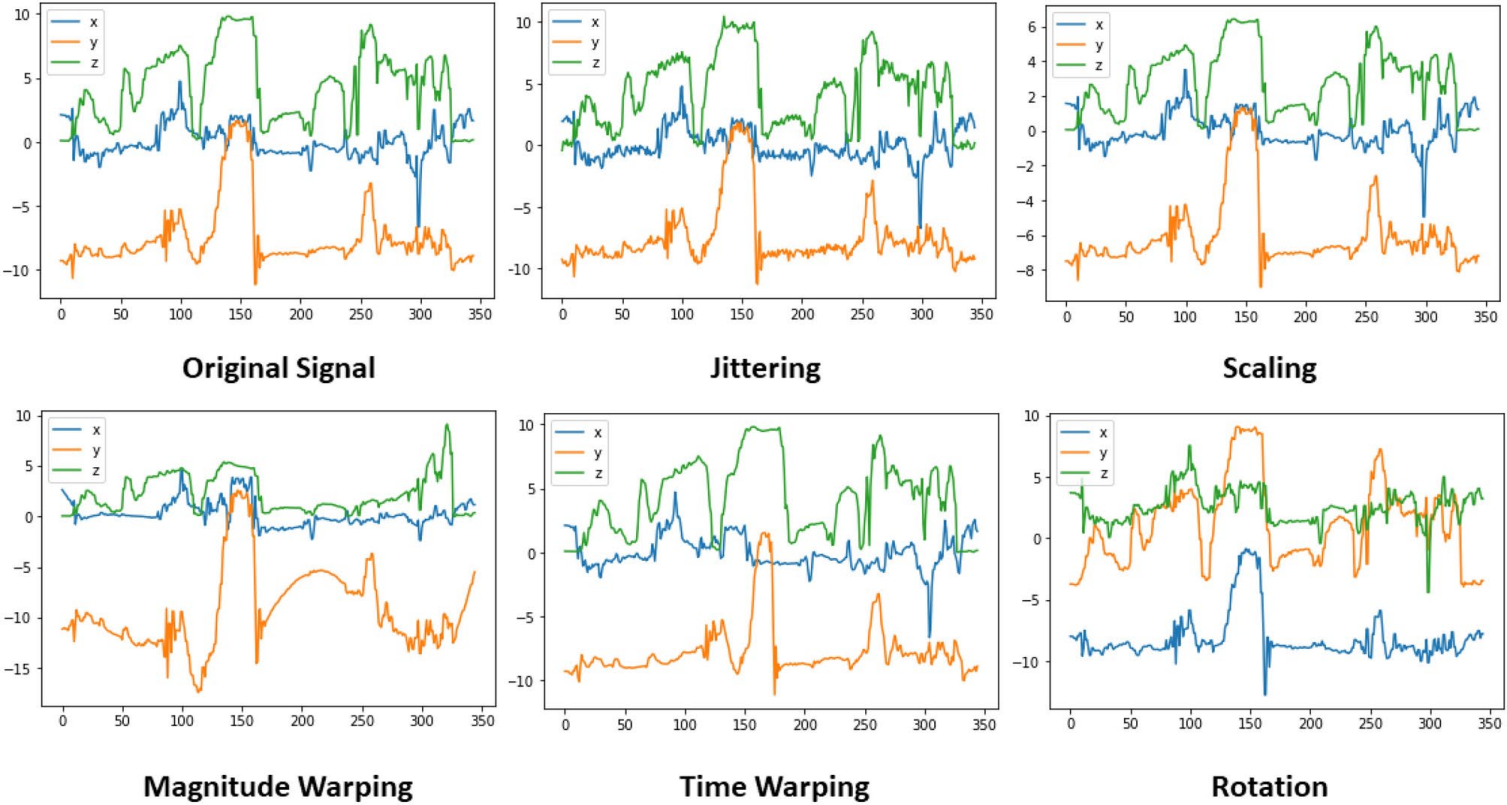
An example of changes in signal pattern with the original for different kinds of augmentation applied to simple random accelerometer hand movement. Here in X-axis is the signal magnitude and Y-axis is the time steps.

The proposed data augmentation procedure is shown in Algorithm 1. It takes the training segments, a variable *A*, and the desired type of augmentation as input. *A* indicates the desired maximum number of segments for each class label. Then, this algorithm groups the segments according to class labels using the function GROUPING, which takes *trainSegments* as input and returns all groups to a variable named *G*. Then it takes each segment of a group and passes it to an augmentation function named AUGMENT with the desired type of augmentation. This process is repeated for each segment of each group until the total number of segments reaches the maximum number of segments denoted by the user as the variable *A*. After augmentation, the augmented segments are appended to their respective groups, and finally, all the groups including augmented and without augmented segments are returned, using a variable named *augG*. In this way, all the classes are balanced having an equal number of segments, which was given as input *A*. 5 types of augmentation and their different combinations are experimented with: jitter, scaling, time Warping (TW), magnitude warping (MW), and rotation (Rot). Figure 3 demonstrates how various data augmentation techniques can alter the original accelerometer signal pattern from a simple hand movement. By applying these transformations, we can generate diverse training data and improve the robustness of our models. The brief descriptions of these augmentation functions are given below:

Jitter is an augmentation function that adds sensor noise to the data and tries to simulate the noisy environment of the real world. It is defined by the following equation,

1 $$\begin{aligned} J(\alpha _{xn})=\alpha _{xn}+Z_{n}, \end{aligned}$$

where, $\alpha _{xn}$ represents the acceleration signal at time step *n* for the x-axis, and $Z_{n}$ is a random variable that takes a random value drawn from a Gaussian distribution. The Gaussian can be expressed by the following equation:

2 $$\begin{aligned} \mathcal {N}(\mu , \sigma )=\frac{1}{\sigma \sqrt{2 \pi }} e^{\frac{1}{2}\left( \frac{x-\mu }{\sigma }\right) ^{2}}. \end{aligned}$$

Like Jitter, scaling is an augmentation method that tries to simulate a noisy environment. But, the only difference is that it simulates multiplicative noise instead of additive noise. It modifies the magnitude of a signal by multiplying it with a random scalar. It can be expressed by the following equation:

3 $$\begin{aligned} S(\alpha _{xn})=\alpha _{xn}*W_{n}, \end{aligned}$$

where, $W_{n}$ is a random variable that takes a random value drawn from the Gaussian distribution.

**Algorithm 1:**
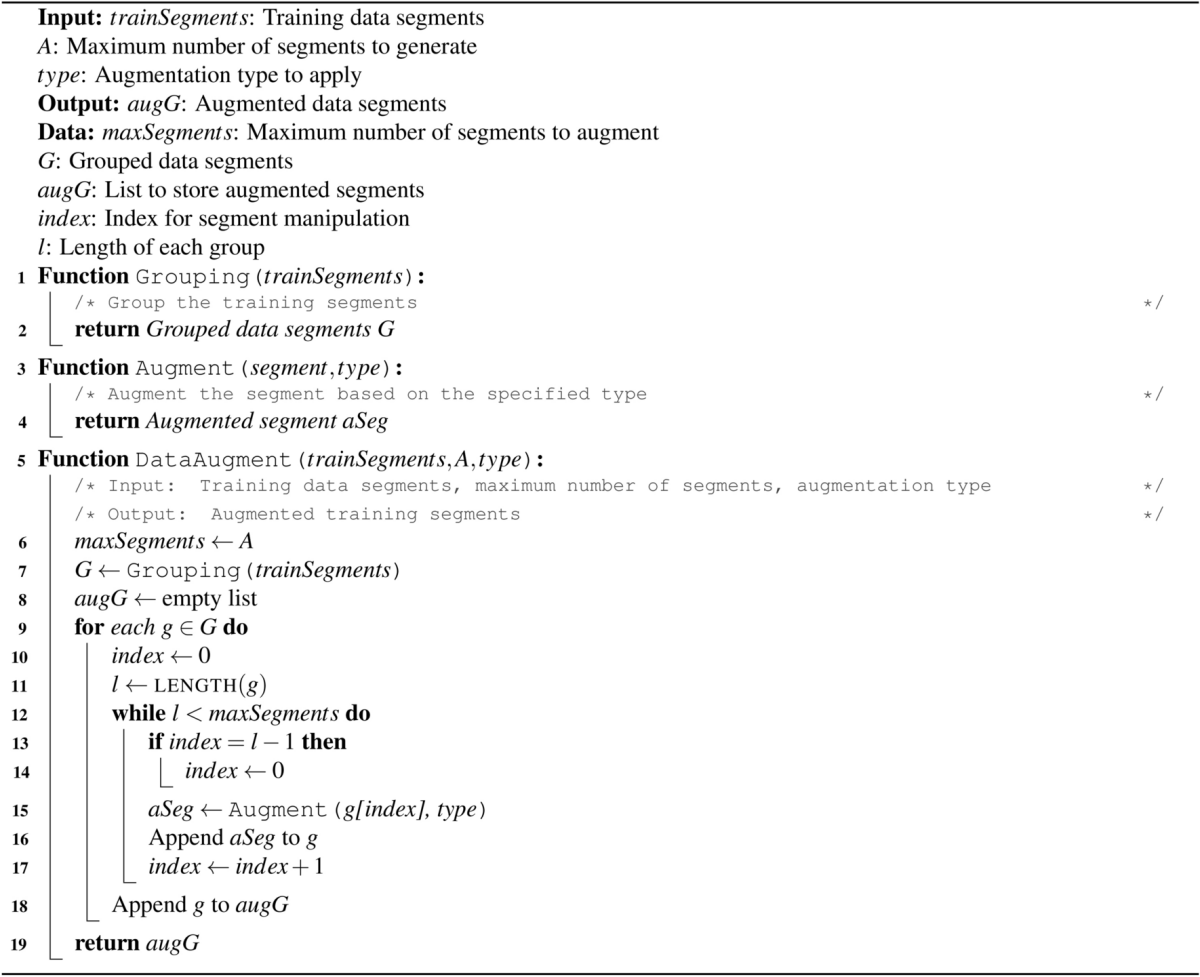
Data augmentation algorithm.

Magnitude warping (MW) perturbs the magnitude of a signal by multiplying it with a random curve which approximately varies around one. For random curve generation, at first, *k* random data points are taken from the Gaussian distribution. This process can be expressed as $Z(k) \sim \mathcal {N}(\mu , \sigma )$, where, *Z*(*k*) is a list of *k* points; and $k = \varphi + 2$; $\varphi$ represents the complexity of the curve. Then, the random curve *r*(*k*) is constructed by cubic spline interpolation which can be expressed as $r:\left[ k_{1}, k_{w+1}\right] \rightarrow \mathbb {R}$, where, *r* is the composition of *w* polynomials of degree 3, referred to as $r_{1}$ to $r_{w}$. Finally, magnitude warping of a signal $\alpha _{x}(n)$ is calculated by the following equation:

4 $$\begin{aligned} MW(\alpha _{x}(n))=\alpha _{x}(n)*r(k). \end{aligned}$$

Time warping (TW) alters the temporal locations of signal samples randomly. It randomly shifts, expands, or compresses the signal temporally. Like in MW, a random curve *r*(*k*) is constructed using cubic spline interpolation. Subsequently, a list of the average cumulative score is calculated by the following equation:

5 $$\begin{aligned} v(k)=\frac{1}{m}\left[ {r(0), \sum _{k=0}^{1}r(k), \sum _{k=0}^{2}r(k),...,\sum _{k=0}^{m-1}r(k)}\right] , \end{aligned}$$

where, *m* is the total number of data points in the actual signal. Finally, for the known points of *v*(*k*) and $\alpha _{x}(n),$ the magnitudes of the signal at points $v = 0, 1, 2,...,m-1$ are calculated using linear interpolation and this step completes all the calculations of TW. So, the TW of a signal $\alpha _{x}(n)$ can be calculated by the following linear interpolation equation:

6 $$\begin{aligned} TW(\alpha _{x}(n))=\sum _{i=0}^{1} \alpha _{x}(n_{i}) \ell _{i}(v), \end{aligned}$$

where, $\ell _{i}(v)$ is the Lagrange basis polynomial and can be calculated by:

7 $$\begin{aligned} \ell _{i}(v)=\prod _{\begin{array}{c} j=0 \\ j \ne i \end{array}}^{1} \frac{v-v_{j}}{v_{i}-v_{j}}. \end{aligned}$$

Rotation (Rot) is an augmentation technique that simulates different sensor orientation and placement conditions. Rotation of the sensor axis can happen when different users wear a sensor at different angles. Rotation of a 3 axis signal is calculated by:

8 $$\begin{aligned} Rot=\left[ \begin{array}{lll}\alpha _{x}(n)&\alpha _{y}(n)&\alpha _{z}(n)\end{array}\right] * Q, \end{aligned}$$

where, *Q* is the rotation matrix calculated from the following equation:

9 $$\begin{aligned} Q=\left[ \begin{array}{ccc} x^{2} c+\cos \theta & x y c-z \sin \theta & x z c+y \sin \theta \\ y x c+z \sin \theta & y^{2} c+\cos \theta & y z c-x \sin \theta \\ z x c-y \sin \theta & z y c+x \sin \theta & z^{2} c+\cos \theta \end{array}\right] , \end{aligned}$$

where, *x*, *y*, and *z* are the components of a unit vector $\hat{u}$ drawn randomly from a uniform distribution of interval [-1, 1]; $c=1 - \cos \theta$; and $\theta$ is an angle drawn randomly from a uniform distribution of interval [$-\pi$, $\pi$]. For both data sets, augmentation is applied before filtering the training data. Finally, the combination of different augmentation functions like the combination of MW and TW is also tested.

### Filtering and intermediate feature set extraction

Despite many precautions, various types of noise were introduced in the raw data. Especially in the field data (taken in the hospital’s challenging environment) of Data Set 2, the noise was more prevalent. The typical bandwidth of a human activity signal is around 0–20 Hz. Hence, we utilize a median filter of window size 5 followed by a low pass Butterworth filter with a cutoff frequency of 20 Hz to eliminate any noise. The order of the Butterworth filter was 3.

To identify different persons with their corresponding activity, meaningful feature extraction from raw data is necessary. For this purpose, we extract the features from the raw data in two steps: One is intermediate feature set (IFS) extraction, and another one is the final feature set (FFS) extraction. The raw accelerometer signal contains three columns of signal $\alpha _{x}$, $\alpha _{y}$, $\alpha _{z}$ from the x, y, and z-axes, respectively. Here, 10 more intermediate feature columns are extracted from these columns. These columns are magnitude (1 column), displacement (3 columns), velocity (3 columns), and angle (3 columns). Finally, both data sets have 13 columns of intermediate features including the raw $\alpha _{x}$, $\alpha _{y}$, $\alpha _{z}$ signals. All definitions of the features of IFS are described in detail in the following paragraphs.

The magnitude of the raw accelerometer signal represents the $L^2$ norm of the acceleration vector. The magnitude of a signal at time step *n* can be illustrated by:

10 $$\begin{aligned} M_n=\sqrt{\alpha _{xn}^{2}+\alpha _{yn}^{2}+\alpha _{zn}^{2}}. \end{aligned}$$

Different users can wear the accelerometer sensor in different orientations and places. This can create large-signal variability for the same activity, which can degrade the performance of any machine learning classifier. Thus, to mitigate this signal variability, the magnitude can be a good feature that creates an orientation-independent representation of the signal.

If we look into the activities, we can see that the orientation angle of the sensors can play a major role in differentiating the activities, and also depending on person-to-person, measure of bending may differ doing even the same activity. As there were only body data present in both data sets, we take a different approach to calculate the angle^48^ rather than opting for gravitation or gyroscope data, which are commonly used for angle calculation in activity recognition. This is done to render our system more robust and be suited for any smart device as many smart devices still do not have a gyroscope, and the calculation of gravitation data is cumbersome. The orientation angle for axis z at time step *n* is calculated by using the following simple formula:

11 $$\begin{aligned} \theta _{zn} = \arctan (\frac{\alpha _{zn}}{\sqrt{\alpha _{xn}^2 + \alpha _{yn}^2}}). \end{aligned}$$

Similarly, the orientation angle $\theta _{xn}$ and $\theta _{yn}$ are also calculated for axes x and y, respectively. Finally, the velocity and displacement of each acceleration axis are calculated by 1st and 2nd order integration, respectively.

### Final feature set extraction

**Fig. 4:**
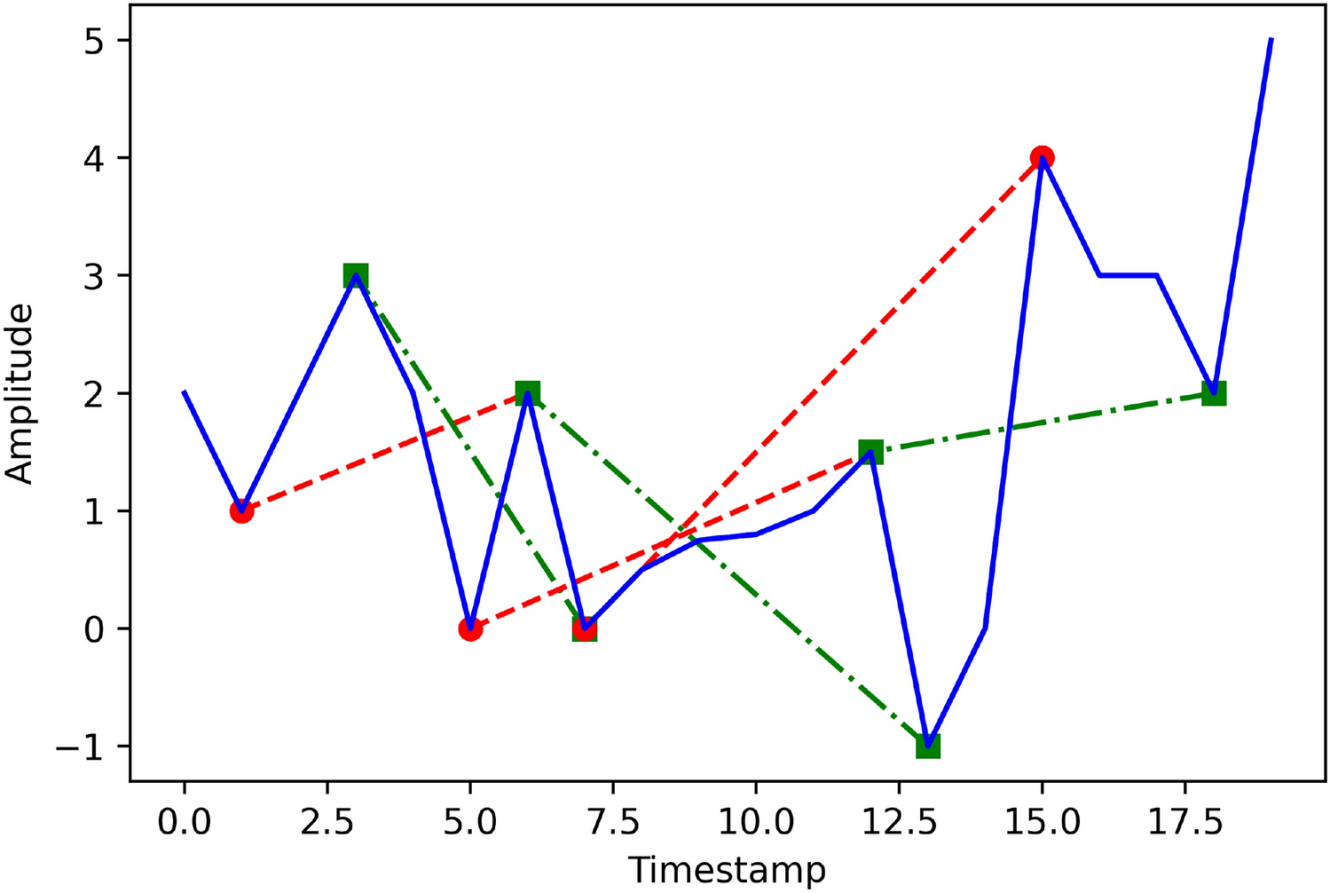
Graphical illustration of the MSUM feature; red(- -) lines indicate distances between relative minima and second successive maxima, green(–.–) lines indicate distances between relative maxima and second successive minima, and blue(–) line indicates the original signal.

For each column of intermediate features, 9 statistical features are calculated, and we name this feature set as Final Feature Set (FFS). So, finally, 13*9 = 117 features are computed. The 9 statistical features are: Standard seviation (SD), average, max, min, variance, median absolute deviation (MAD), mean min max sum (MSUM), energy, and interquartile range (IQR). Here, with the commonly used statistical features, we propose a new feature named MSUM. For calculating this feature, we first locate all the relative maxima and minima of a signal. Then, the distance between relative maxima and their second successive minima is calculated. Similarly, the distance between relative minima and their second successive maxima is extracted. Finally, all the distances are summed and divided by the total number of lines to compute the average distance. In this way, MSUM is calculated. Figure 4 shows the graphical illustration of this feature. From this figure, the feature can be calculated by:

12 $$\begin{aligned} MSUM=\frac{\sum _{j=0}^{{R}N-1}\left| {R}_{fj}-{R}_{ij}\right| +\sum _{j=0}^{{G}N-1}\left| {G}_{fj}-{G}_{ij}\right| }{N}, \end{aligned}$$

where, ${R}_f$ and ${R}_i$ indicate the final and initial points of the red lines; ${G}_f$ and ${G}_i$ indicate the final and initial points of green lines; *RN* and *GN* are the total numbers of red and green lines respectively; and $N = {R}N+{G}N.$ Inspired by^49^, we designed this feature. The difference is that^49^ use the first successive maxima and minima, but we use the second successive maxima and minima. As complex nursing activities are composed of many simple activities, this feature can be useful for calculating the temporal relationships of those simple activities. The reason behind considering the second successive maxima and minima is that in this way, one can find not only short temporal relations, but also long temporal relations of simple activities. This feature can also replace max, min, and mean features alone.

### Feature selection

To precisely identify and systemically analyze the activities, feature selection plays an important role. Not all the features bear equal importance, and some may even be superfluous and irrelevant, which may lead to overfitting or poor accuracy. Thus, to select the best features, the correlations of all features are calculated, and highly correlated features are eliminated from the correlated pairs. As correlation coefficient, we employ the Pearson correlation coefficient. After eliminating the highly correlated features, the features are ranked according to the importance factor of a random forest (RnF) classifier. Here, RnF is chosen because it showed the best performance in the initial assessment (i.e., compared with boosted tree, tree, quadratic support vector machine (QSVM), and K-Nearest Neighbor (KNN)).

### Proposed stacked CNN (S-CNN) model

**Fig. 5:**
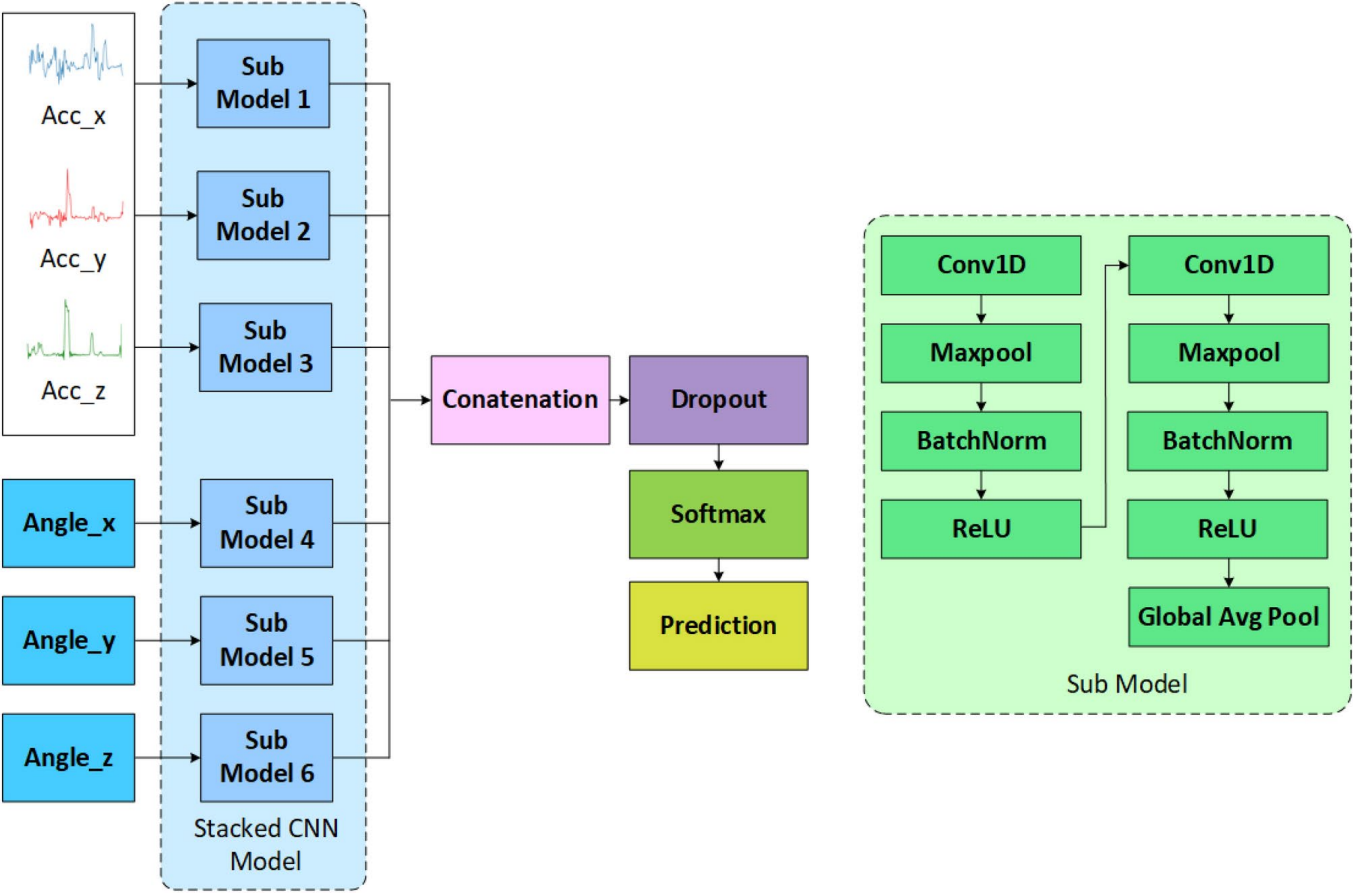
The proposed stacked CNN (S-CNN) model, which takes acceleration and angle inputs of the x, y, and z axes.

Figure 5 shows the proposed Stacked CNN (S-CNN) model. In a traditional CNN, all the input channels are taken together but in a S-CNN, the input channels are taken separately in different paths and in different sub-models. Here, along with the acceleration inputs of the x, y, z axes, we also consider the angles calculated for each axis. Angles act as important distinctive features as activities were conducted in different angles. We also experimented with other intermediate features such as magnitude, displacement, velocity, but our analysis showed that those features do not act as distinctive features in this case. We also conducted an experiment with traditional CNN using these same (Acc_xyz and Angle_xyz) features, yet, leading to low performance. The reason is that acceleration and angles are quite dissimilar features requiring different paths in the initial stage of the model. Hence, the S-CNN approach is more suitable in this case. As sub-model, we use two layers of 1D convolution, maxpooling of size 2, batch normalization (BatchNorm), and a Rectified Linear Unit (ReLU) activation function. We choose such a shallow model to avoid overfitting. All the sub-models are concatenated followed by a dropout layer (*p* = 0.25) and a softmax layer. The first convolution layer has 32 filters of kernel size = 16, and the second convolution layer has 16 filters of kernel size = 16. The hyperparameters are chosen using a random search algorithm. The model is trained using the Adam optimizer, a learning rate = 0.001, batch size = 32, an epoch number = 500, and the loss function = categorical cross-entropy. The training is stopped if the validation accuracy does not improve for 100 epochs. To build the ensemble model and decision fusion of the classical machine learning model and the S-CNN model, we take the weighted sum of the predicted probabilities of each model using:

**Algorithm 2:**
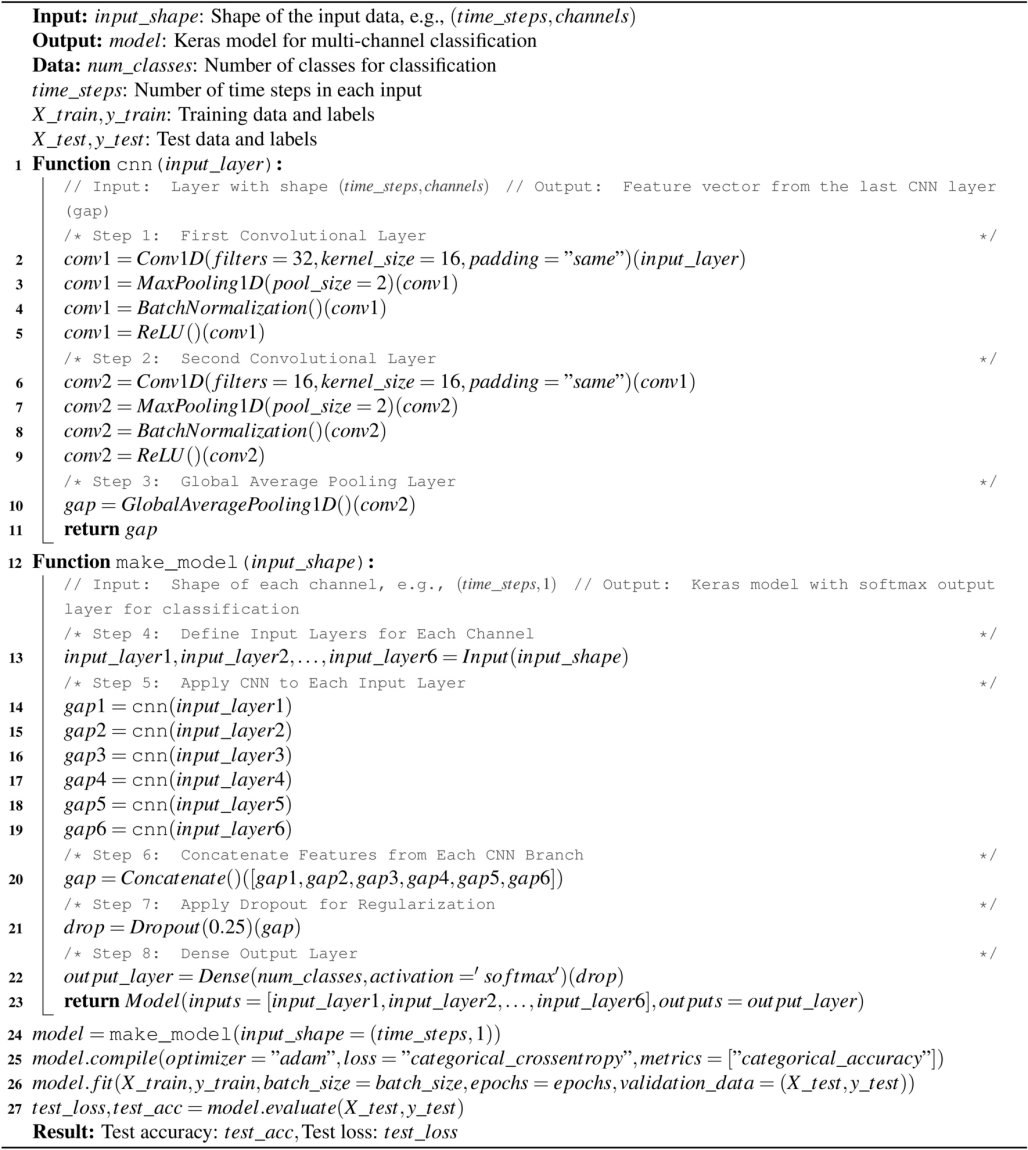
Stacked CNN.

13 $$\begin{aligned} S_{\textrm{ws}}(v=y)=\sum _{i=1}^{n} \beta _{i} P_{i}\left( m_{i}=y\right) , \end{aligned}$$

where, *n* is the number of models used to classify a sample *v*, $P_{i}\left( m_{i}=y\right)$ is the probability of assigning the activity *y* to the activity label in model $m_{i}$, and $\beta _{i}$ is the weight associated with the model $m_{i}$. $\beta$ values were selected empirically during the validation stage. After summation, the class with the highest probability is taken as the output label. The pseudocode for this model is shown in Algorithm  2. 

For some hyperparameters, we started with default values as initial benchmarks. We employed a combination of random search and cross-validation techniques to methodically explore a range of values for each hyperparameter. We tested hyperparameter ranges as follows: learning rate (0.0001, 0.001, 0.01), batch size (32, 64, 128), and epoch number (100, 200, 300, 400, 500).

### Technical details

The technical details of our work are:

1. Device Specification: RAM: 32GB, CPU: Intel(R) Xenon(R) E52690 @3GHz(16cpus)
2. Programming Languages: Python 3.11
3. Libraries: Classical machine learning and deep learning pipeline were built using Scikit-learn 1.5.2^50^ and tensorflow 2.18.0^51^ respectively. Data processing was done using numpy 2.0.0^52^ and pandas 2.2.3^53^. Figures were generated using Matplotlib 3.9^54^.

## Result and discussion

In this section, all the experimental results and analyses will be discussed. For performance evaluation, we use accuracy, precision, recall, and F1 score as performance metrics. A detailed analysis of both activity and nurse identification is given.

**Fig. 6:**
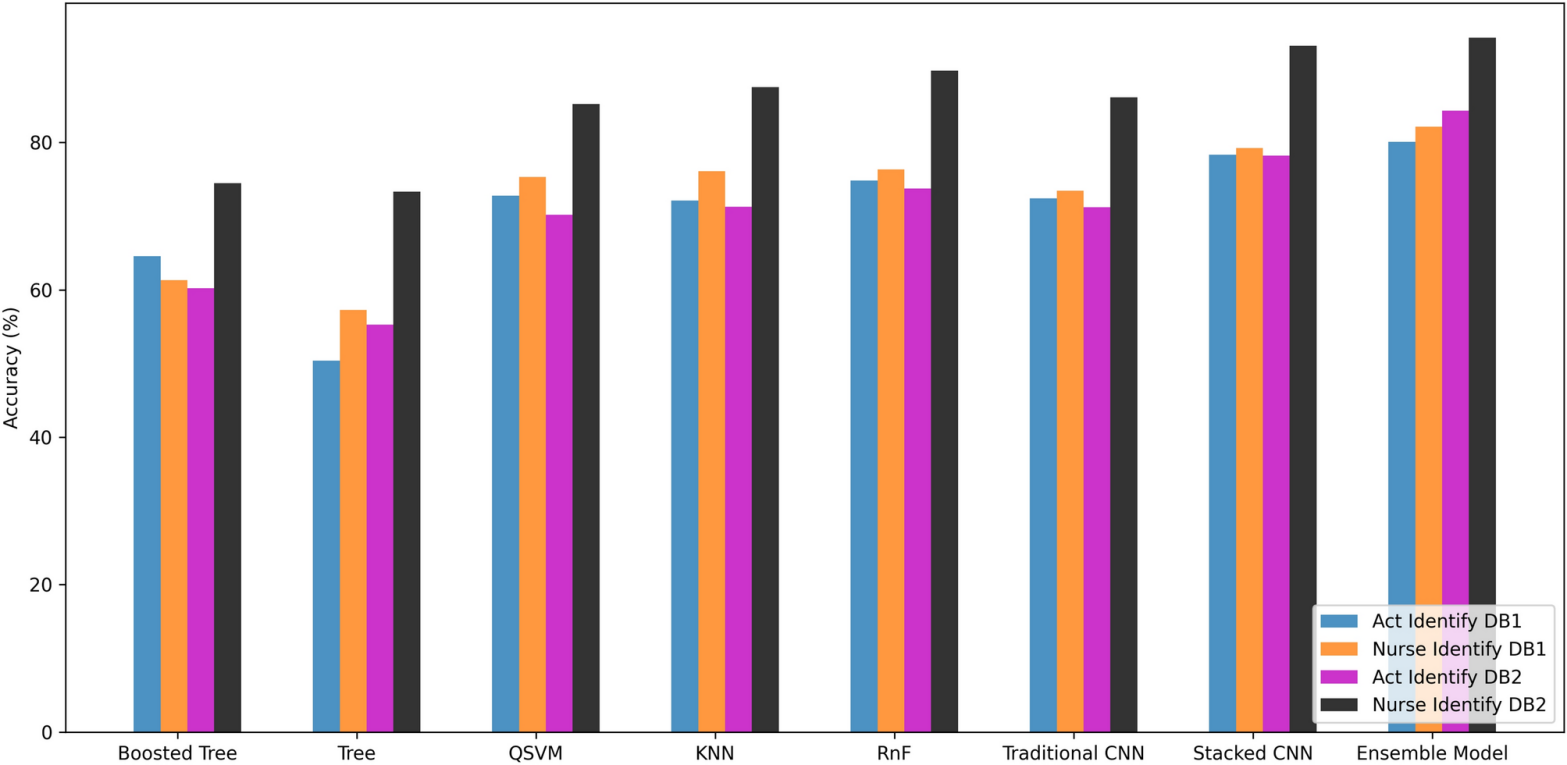
Comparison of the top 5 algorithms on Data Set 1 and 2 for both activity and nurse identification.

### Overall analysis

At first, different machine learning classifiers are tested. Note that, an ensemble model performed best in every experiment, which can be seen in Fig. 6. Leave-one-subject-out cross validation (LOSOCV) has been taken as the standard procedure. Figure 6 shows the comparison of average validation accuracies of the top 8 algorithms. For Data Set 1, the ensemble model achieves the highest accuracies of 80.1% and 82.1% for activity and nurse identification, respectively. For Data Set 2, the ensemble model also achieves the highest accuracies of 84.3% and 94.2% for activity and nurse identification, respectively. During decision fusion, a higher weight factor ($\beta$ = 2 for S-CNN) and lower weight factor ($\beta = 1$ for RnF) gave the best results.

**Fig. 7:**
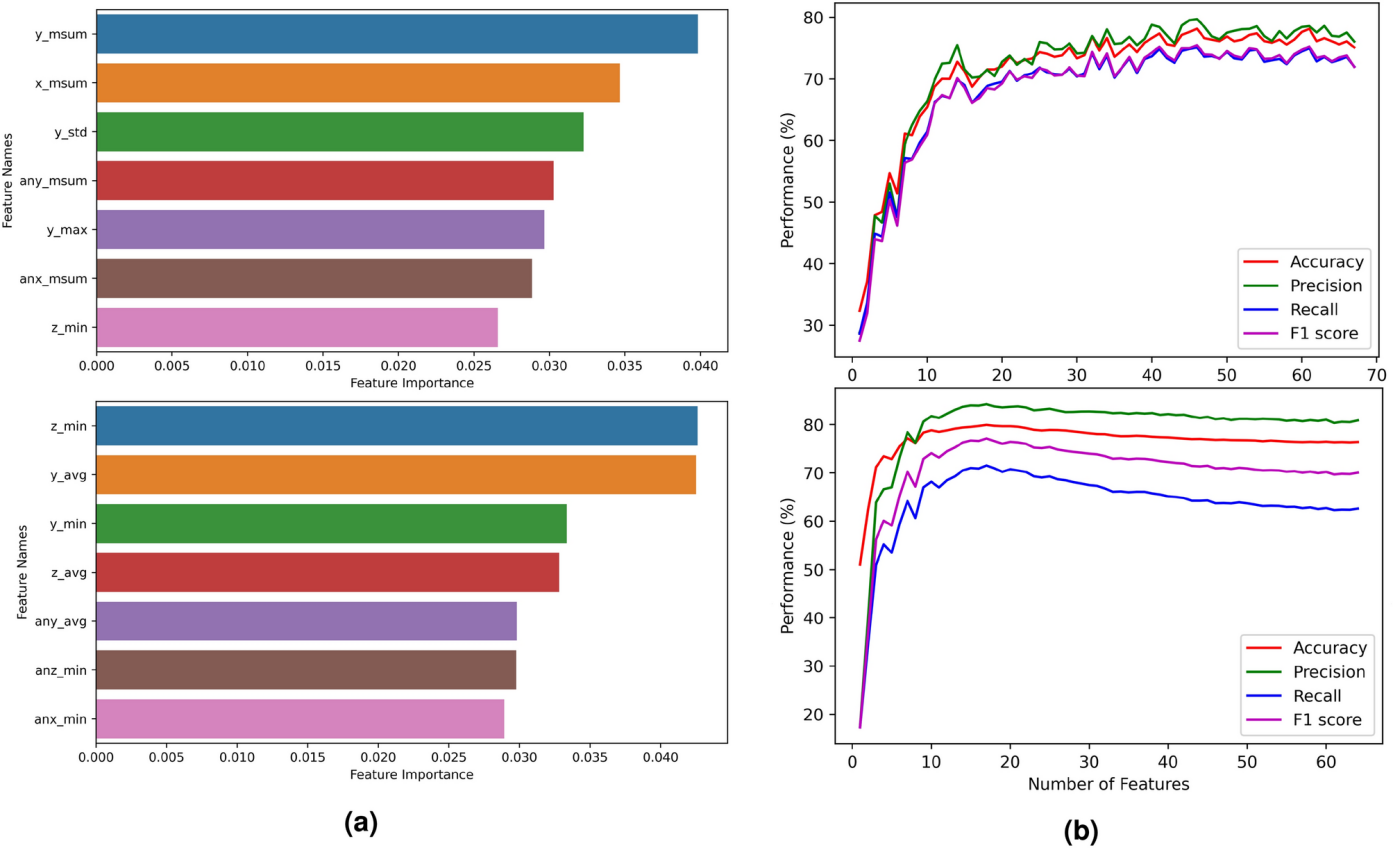
Feature based analysis: (**a**) comparison of the most important features of the activity recognition system on Data Set 1 (left top) and 2 (left bottom) and (**b**) Performance curve of the activity recognition system on Data Set 1 (right top) and 2 (right bottom) based on the number of features used.

After testing with all features, we rank the features according to MDI for both data sets, and the feature importance scores of the top 7 features for activity recognition in the Data Set 1 and 2 can be seen in Fig. 7a. After ranking, performances are evaluated by increasing the features gradually, and a performance vs feature graph is drawn. Figure 7b shows such a graph for both data sets. From this figure, it can be observed that after achieving a certain accuracy, the curve remains saturated even if the features are increased. This saturation point is taken as the final point for selecting the best features. In this way, 46 and 17 features are finally selected for the activity recognition system in Data Set 1 and 2, respectively. For the user identification system, 37 and 16 features are selected for Data Set 1 and 2, respectively. Lastly, these feature-selected tables are given as input to several classifiers, and the best performing classifier is chosen as the final model. In Fig. 7a, we can also see that our proposed feature MSUM coupled with the acceleration of the y-axis (y_msum) has achieved the highest importance score in Data Set 1, and most of the features in the top seven features are also related to MSUM. This clearly shows the appropriateness of our proposed feature for nursing activity recognition on Data Set 1. For Data Set 2, the most important feature for activity recognition is the minimum value of the z-axis (z_min) acceleration. Similarly, for nurse identification, the most important features are z_min and the average value of z-axis (z_avg) acceleration on Data Set 1 and 2, respectively.

Then, to tackle the class imbalance problem, different types of data augmentations have been applied. Table 1 shows the performance of our activity recognition system with and without data augmentation on Data Set 1 and 2. The highest value in a row is marked with a bold number. In terms of LOSOCV accuracy, the highest accuracy is achieved by TW augmentation with 78.7% accuracy. This is 3.8% higher than the accuracy without augmentation, which is 74.9%. In terms of recall and F1 score, the highest values are also observed for TW with the values of 78.4% and 77.7%, respectively. These values are also 6.2% and 5.6% higher than the values without data augmentation. On Data Set 2, small improvement is seen in terms of overall accuracy, precision, and recall. In this case, the highest values are also observed for TW augmentation. We also experimented with different amount of augmentation and found that augmenting the original data set by 3 times produced the best results.

**Table 1:** Comparison of various augmentation techniques for the activity recognition system.

Performance metrics (%)	Without augmentation	Jitter	Scaling	MW	TW	Rot	MW and TW
*Data Set 1*							
Accuracy	74.9	77.6	76.9	77.9	**78.7**	74.6	76.9
Precision	76.2	78.7	76.9	**79.2**	78.6	76.3	78.5
Recall	72.2	77.4	75.3	76.5	**78.4**	73.4	77.8
F1 score	72.1	76.6	74.7	76.3	**77.7**	72.8	76.3
*Data Set 2*							
Accuracy	73.7	73.7	73.6	73.5	**74.0**	73.6	73.5
Precision	77.2	77.2	77.2	76.1	**77.6**	76.3	76.4
Recall	56.2	56.4	56.2	54.2	**56.4**	54.2	55.9
F1 score	65.0	64.9	64.9	63.8	**65.8**	63.3	63.1

The highest values are in bold.

Table 2 shows the performance comparison of the activity recognition system for different intermediate feature sets on Data Set 1. Table 3 depicts the similar comparison for Data Set 2. From these tables, it is evident that for RnF, the intermediate feature set of Acc_xyz, Magnitude, Displacement, Velocity, and Angle achieves the highest accuracy, precision, recall, and F1 score. For Data Set 1 and RnF, the highest achieved accuracy, precision, recall, and F1 score are 78.7%, 78.6%, 78.4%, and 77.7%, respectively. For Data Set 2 and RnF, the highest achieved accuracy, precision, recall, and F1 score are 74.0%, 77.6%. 56.4%, and 65.8% respectively. Both RnF and S-CNN performed better when the angle was given as intermediate features. For the S-CNN, highest performance is achieved using the combination of Acc_xyz and angle. For Data Set 1 and the S-CNN, the highest achieved accuracy, precision, recall and F1 score are 80.1%, 81.3%, 81.2%, and 81.2%, respectively. For Data Set 2 and the S-CNN, the highest achieved accuracy, precision, recall and F1 score are 84.3%, 83.2%, 82.2%, and 82.6%, respectively. These performances were achieved due to the contribution of the angle, which is evident from the comparison with other intermediate features. It appears clear that the ‘traditional’ CNN performs worse than RnF and the S-CNN in almost all combinations, which indicates its low suitability in these cases.

**Table 2:** Performance evaluation of the activity recognition system for different intermediate feature sets on Data Set 1.

Model	Intermediate features	Accuracy (%)	Precision (%)	Recall (%)	F1 score (%)
RnF	Acc_xyz	72.8	72.3	72.6	72.4
	Acc_xyz, Magnitude	72.5	72.4	72.9	72.7
	Acc_xyz, Magnitude, displacement	74.3	73.5	74.2	73.7
	Acc_xyz, Magnitude, dispalcement, velocity	73.2	72.9	73.4	73.1
	Acc_xyz, Magnitude, dispalcement, velocity, angle	78.7	78.6	78.4	77.7
Traditional CNN	Acc_xyz	74.2	74.5	74.6	74.5
	Acc_xyz, Magnitude	72.3	71.6	71.7	71.6
	Acc_xyz, Magnitude, displacement	70.6	70.2	70.4	70.2
	Acc_xyz, Magnitude, dispalcement, velocity	68.9	69.1	69.2	69.1
	Acc_xyz, Magnitude, dispalcement, velocity, angle	71.2	71.8	71.5	71.7
	Acc_xyz, Angle	72.4	73.2	73.6	73.5
S-CNN	Acc_xyz	74.5	74.6	74.5	74.5
	Angle	77.3	77.6	77.4	77.5
	Acc_xyz, Magnitude	75.2	75.1	75.6	75.4
	Acc_xyz, Displacement	76.4	76.1	76.2	76.1
	Acc_xyz, Velocity	74.8	74.9	74.8	74.8
	Acc_xyz, Magnitude, dispalcement, velocity, angle	76.3	77.5	77.2	77.3
	Acc_xyz, Angle	80.1	81.3	81.2	81.2
Ensemble model	Magnitude, displacement, velocity,angle	84.2	85.1	85.3	85.2

**Table 3:** Performance evaluation of the activity recognition system for different intermediate feature sets on Data Set 2.

Model	Intermediate features	Accuracy (%)	Precision (%)	Recall (%)	F1 score (%)
RnF	Acc_xyz	68.1	72.1	51.3	61.5
	Acc_xyz, Magnitude	67.3	71.2	51.2	61.7
	Acc_xyz, Magnitude, displacement	69.5	73.2	53.2	63.2
	Acc_xyz, Magnitude, dispalcement, velocity	68.2	72.3	52.1	61.2
	Acc_xyz, Magnitude, dispalcement, velocity, angle	74.0	77.6	56.4	65.8
Traditional CNN	Acc_xyz	73.2	74.5	54.6	65.2
	Acc_xyz, Magnitude	74.3	73.6	55.7	63.2
	Acc_xyz, Magnitude, displacement	72.6	70.7	58.2	66.4
	Acc_xyz, Magnitude, dispalcement, velocity	71.9	74.2	59.2	67.8
	Acc_xyz, Magnitude, dispalcement, velocity, angle	75.7	71.7	54.2	65.2
	Acc_xyz, Angle	72.7	73.2	57.2	67.9
S-CNN	Acc_xyz	79.5	79.6	79.5	79.5
	Angle	81.3	81.1	82.2	81.6
	Acc_xyz, Magnitude	77.2	78.1	76.6	77.4
	Acc_xyz, Displacement	80.4	81.6	81.8	81.4
	Acc_xyz, Velocity	76.8	76.3	74.8	75.6
	Acc_xyz, Magnitude, dispalcement, velocity, angle	78.3	79.5	79.2	79.3
	Acc_xyz, Angle	84.3	83.2	82.2	82.6
Ensemble model	Magnitude, dispalcement, velocity, angle	87.2	87.5	87.1	87.3

**Table 4:** Performance evaluation of the activity recognition system with and without feature selection.

Performance metrics (%)	Without feature selection	With feature selection	With feature selection and TW augmentation
*Data Set 1*			
Accuracy	74.9	78.2	79.4
Precision	76.1	79.7	80.6
Recall	72.0	75.1	79.3
F1 score	72.1	75.5	78.8
*Data Set 2*			
Accuracy	73.7	79.5	79.7
Precision	77.2	83.4	84.4
Recall	56.2	70.4	70.5
F1 score	65.0	76.1	76.2

**Table 5:** Performance evaluation of the nurse identification system with and without feature selection.

Performance metrics (%)	Without feature selection	With feature selection	With feature selection and TW augmentation
*Data Set 1*			
Accuracy	76.4	**78.2**	**78.2**
Precision	80.4	**80.9**	**80.9**
Recall	75.4	77.6	**77.7**
F1 score	75.0	**77.1**	76.9
*Data Set 2*			
Accuracy	89.8	**92.7**	91.9
Precision	90.2	**92.6**	91.7
Recall	76.1	81.9	**82.0**
F1 score	81.2	**86.4**	86.3

The highest values are in bold.

As TW gives the best results, further experiments have been carried out using this augmentation. Table 4 shows the results of feature selection combined with augmentation for activity recognition on both data sets. For Data Sets 1 and 2, the highest accuracy is observed with only 46 features and 17 features, respectively. Combining with augmentation, the highest accuracy for Data Sets 1 and 2 is 79.4%, and 79.7%, respectively. From this experiment, it is evident that feature selection improves performance more than augmentation.

User (Nurse) wise activity recognition performance is next analyzed. There are variations in recognition accuracy, which proves that our system is highly user-dependent. The highest accuracy has been achieved for user 7 with the value of 99.4% accuracy on Data Set 2. Like activity recognition, the same experiments have been carried out for the user identification system. Table 5 shows the results of our user identification system with feature selection and augmentation. For Data Sets 1 and 2, the best accuracy – 78.2% (using 37 features), and 92.7% (using 16 features) is achieved, respectively.

**Table 6:** Comparative analysis for Data Set 1.

Method	Training accuracy (%)	Testing accuracy (%)
Raw data and RnF^29^	82.9	18.1
Basic statistical features and RnF^15^	60.0	43.1
Intermediate features (Acc_xyz, angle,magnitude,displacement, and velocity), Statistical features (SD, average, max,min, variance, MAD, MSUM, energy, and IQR), and RnF	79.4	62.1
Intermediate features (Acc_xyz, Angle) and S-CNN	83.2	68.1
All intermediate, statistical features and ensemble model	85.6	70.6

**Table 7:** Comparative analysis for Data Set 2.

Method			Training accuracy (%)	Testing accuracy (%)
Features	Window size	Classifier		
Raw tri-axial acceleration signals^36^	10	Deep-LSTM	97.4	0.2
Raw Data^33^	1	CNN	91.6	0.8
Signal Energy, Mean, Auto- correlation, SD, RMS, SMA, and Spectral Entropy^32^		RnF	78.3	1.3
Min, Max, SD, Mean, Variance, Peak to Peak Range, (Max, average and SD) of Rate of Change, MAD, IQR, Autocorrelation, Mean Crossing Rate, Linear Velocity, Kinetic Energy, SMA, Max Power^31^	1000	RnF	78.0	10.6
Mean, Variance, Median, Percentile25, Percentile75, Min, Max, Skewness, Kurtosis, Signal power, Covariance^55^	8	RnF	48.3	12.3
SD, Max, Min, Variance, MAD, Mean, Energy, and IQR^34^	128	RnF	65.9	15.5
Mean, SD, MAD, Max, Min, Signal magnitude, Energy, IQR, Signal entropy, Correlation, Skewness, Kurtosis, MaxInds^35^		RnF	74.5	19.4
Mean, Median, Mode, Variance, SD and RMS of coordinates^37^	60	KNN	75.0	22.4
Intermediate Features (Acc_xyz, Angle, Magnitude, Displacement, and Velocity), and Statistical Features (SD, Average, Max, Min, Variance, MAD, MSUM, Energy, and IQR)	60	RnF	79.7	69.5
Intermediate Features (Acc_xyz, Angle)	60	Stacked CNN	87.4	82.3
All intermediate and statistical features	60	Ensemble model	89.4	85.7

Finally, our proposed activity recognition system is tested using the separate test data set provided by the challenge organizer. Table 6 shows the comparison of our method with other methods applied to the test Data Set 1, and Table 7 shows the comparison for test Data Set 2. In both cases, our proposed ensemble model achieves the highest test accuracies of 70.6% and 85.7%, which outperform the other methods with a considerable margin. From the comparison, it is evident that the ensemble model achieved 8.5% and 16.2% higher accuracy than RnF on Data Set 1 and 2, respectively. The ensemble model achieved 2.5% and 3.4% higher accuracy than the S-CNN on Data Set 1 and 2, respectively. The possible reason for such high accuracy is the proposed two-step (Intermediate Feature Set and Final Feature Set) feature extraction method coupled with our proposed S-CNN model. Also, the ensemble of the ‘classical’ RnF with the S-CNN boosts the accuracy. Another possible reasons can be the introduction of the angle as intermediate feature. Angle appears as a suitable feature for nursing activity recognition. It is also evident from the difference in training and test accuracies that our method has reduced the overfitting problem considerably as compared to other methods.

**Fig. 8:**
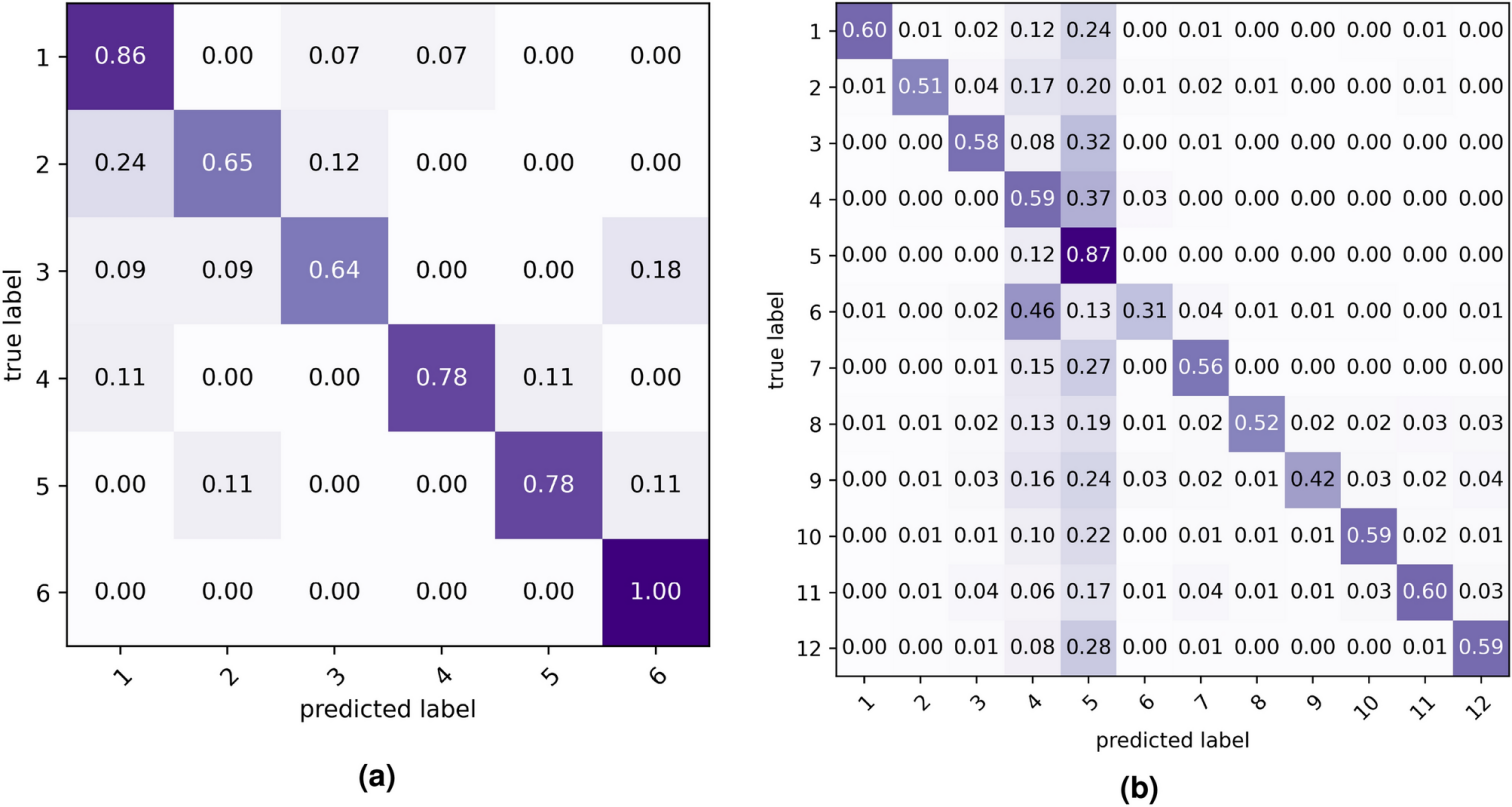
(**a**) Confusion Matrix of the activity recognition system on Data Set 1 and (**b**) Confusion Matrix of the activity recognition system on Data Set 2.

Figure 8a and b show the Confusion Matrices of the activity recognition system on Data Set 1 and 2, respectively. From Fig. 8a, it is evident that the best-detected activity is ‘Diaper Exchange and Cleaning of the Area’ (A6), and the worst is ‘Blood Glucose Measurement’ (A3). The most confusing activities are ‘Blood Collection’ (A2), and ‘Blood Glucose Measurement’ (A3) – as they are quite similar. For Data Set 2, the most confusing activities are ‘Wheelchair’ (A4) and ‘All Assistance’ (A5) (Fig. 8b). In fact, ‘All Assistance’ (A5) is an activity, which has similarities with most of the activities. This can be understood intuitively and can be seen in the Confusion Matrices.

### Ablation study

To gain deeper insights into the contributions of individual components of our proposed model, we conducted an ablation study on two datasets: Data Set 1 and Data Set 2. The results are summarized in Tables 8, 9 and 10 respectively. Key findings from the ablation study are:

1. Effectiveness of S-CNN: The S-CNN consistently outperformed both the RnF and Traditional CNN models on both datasets. This highlights the advantage of using separate sub-models for different input features, allowing the network to learn more discriminative representations.
2. Ensemble Learning: The Ensemble Model further improved performance by combining the predictions of multiple models. This suggests that combining diverse models can lead to more robust and accurate predictions.
3. Similar Test Runtime: We can see that all the models have similar runtime. The reason behind this is because the test data is comparatively smaller in size.

**Table 8:** Ablation study for Data Set 1.

Model	Accuracy (%)	Precision (%)	Recall (%)	F1 score (%)
RnF	78.8	78.4	78.6	78.5
Traditional CNN	74.2	74.5	74.7	74.6
S-CNN	81.4	81.2	80.5	80.8
Ensemble model	85.6	84.9	85.2	85.0

**Table 9:** Ablation study for Data Set 2.

Model	Accuracy (%)	Precision (%)	Recall (%)	F1 score (%)
RnF	74.5	77.8	57.4	66.1
Traditional CNN	74.2	74.5	55.6	63.7
S-CNN	84.4	83.2	82.5	82.8
Ensemble model	89.4	88.9	89.2	89.0

**Table 10:** Ablation study based on time.

Model	Testing time (s) Data Set 1	Testing time (s) Data Set 2
RnF	14.7	25.4
Traditional CNN	16.9	28.7
S-CNN	19.8	30.2
Ensemble model	22.3	32.7

### Comparative analysis with related works

Table 11 presents a comparative analysis of our proposed method with other state-of-the-art approaches. Our method achieved superior performance on both Data Set 1 and Data Set 2, particularly in terms of testing accuracy. This significant improvement can be attributed to the effectiveness of the S-CNN architecture and the integration of relevant features.

**Table 11:** Performance comparison with other works.

Works	Training accuracy (%)	Testing accuracy (%)
*Data Set 1*		
Baseline ML^15^	60.00	43.10
Data Digger^29^	82.86	18.10
Our proposed method	85.60	75.60
*Data Set 2*		
Hex Code^31^	78.00	10.58
Gudetama^32^	78.33	01.25
DataDrivers_BD^33^	91.59	00.76
UCLab^55^	48.30	12.26
Britter Baire^34^	65.90	15.53
Team Apophis^35^	74.50	19.39
HealthyVibes^36^	97.40	00.17
MoonShot_BD^37^	75.00	22.35
Our proposed method	89.40	85.70

The results of the ablation study and performance comparison demonstrate the effectiveness of our proposed approach for activity recognition in elderly care. The S-CNN architecture, combined with ensemble learning, provides a robust and accurate solution to this challenging problem.

## Conclusion and future work

In this paper, we have shown a single robust system to identify users and activities in the real world. From our analyses, it is clear that intermediate features combined with a sophisticated Stacked CNN approach can achieve good results in complex activity recognition even on imbalanced data sets. Unlike traditional CNN, the Stacked CNN takes different sensor branches parallelly and integrates different information sources more accurately. Overfitting is a major problem in any machine learning classification algorithm. Our proposed feature extraction and selection method combined with the data augmentation algorithm has reduced this problem considerably. It is interesting to note that Time Warping (TW) augmentation has given the best results in most cases. As the tasks of activity recognition and user identification are different, the important features are also different. Our proposed method can detect these features and rank them automatically.

Though our method achieves higher accuracy compared to other methods, there remains a demand for improvement. The proposed data augmentation method has improved the performance little. Hence, a more robust augmentation method should be utilized. Possible solutions can be using more sophisticated generative models like variational autoencoders (VAE)^56^, generative adversarial networks (GAN)^57^, Diffusion models^58^. More robust latent feature extraction methods for handling diverse and complex nursing activity (with different contextual factors, sensor noise, intra-class variability) should be searched for better performance. A promising direction is use of Large Language models suitable for IMU based human activity recognition^59^. In the future, we would also like to predict future activity along with identifying the current activity and the user.

## Data Availability

Data sets can be found in the IEEE DataPort repository: https://ieee-dataport.org/competitions/nurse-care-activity-recognition-challenge? and https://ieee-dataport.org/open-access/nurse-care-activities-datasets-laboratory-and-real-field?.
